# Co-creating community support strategies for chronic disease management: insights from our experience with two community groups in southern Quito, Ecuador

**DOI:** 10.1093/heapro/daag123

**Published:** 2026-08-18

**Authors:** Marta Puig-García, María José Sanchís-Ramón, Elisa Chilet-Rosell, Sergio Morales-Garzón, Lucy Anne Parker

**Affiliations:** Department of Public Health, History of Science and Gynaecology, Universidad Miguel Hernández de Elche, Crta. Nacional, N-332, s/n, 03550 Sant Joan d'Alacant (Alicante), Spain; CIBER de Epidemiología y Salud Pública (CIBERESP), Avenida Montforte de Lemos, 3-5 (Pabellón 11, Planta 0), 28029 Madrid, Spain; Department of Public Health, History of Science and Gynaecology, Universidad Miguel Hernández de Elche, Crta. Nacional, N-332, s/n, 03550 Sant Joan d'Alacant (Alicante), Spain; CIBER de Epidemiología y Salud Pública (CIBERESP), Avenida Montforte de Lemos, 3-5 (Pabellón 11, Planta 0), 28029 Madrid, Spain; Department of Public Health, History of Science and Gynaecology, Universidad Miguel Hernández de Elche, Crta. Nacional, N-332, s/n, 03550 Sant Joan d'Alacant (Alicante), Spain; CIBER de Epidemiología y Salud Pública (CIBERESP), Avenida Montforte de Lemos, 3-5 (Pabellón 11, Planta 0), 28029 Madrid, Spain; Department of Public Health, History of Science and Gynaecology, Universidad Miguel Hernández de Elche, Crta. Nacional, N-332, s/n, 03550 Sant Joan d'Alacant (Alicante), Spain; Department of Public Health, History of Science and Gynaecology, Universidad Miguel Hernández de Elche, Crta. Nacional, N-332, s/n, 03550 Sant Joan d'Alacant (Alicante), Spain; CIBER de Epidemiología y Salud Pública (CIBERESP), Avenida Montforte de Lemos, 3-5 (Pabellón 11, Planta 0), 28029 Madrid, Spain

**Keywords:** participatory research, chronic illness, type 2 diabetes, social support, community health promotion, Latin America

## Abstract

Social support plays an important role in the management of type 2 diabetes (T2DM), yet health promotion strategies often focus on individual behavior change while overlooking community structures that sustain supportive environments and facilitate adherence to healthy behaviors. Participatory approaches can help identify locally grounded strategies to strengthen community support networks for chronic disease management. This study aimed to co-create community-based strategies to strengthen social support networks for T2DM management. Between March and September 2024, we conducted a participatory research process with two communities in southern Quito, Ecuador. A core group composed of community leaders and academic representatives facilitated a three-phase process including situational analysis, co-creation of strategies, evaluation, and dissemination. Four community workshops engaged 152 participants. Proposed actions were prioritized through a weighted scoring system assessing feasibility, community ownership, and potential impact on social support networks. Participants generated 15 community-driven proposals. The highest-ranked proposal was the creation of a community health committee responsible for identifying socially isolated individuals, raising family awareness, and facilitating accompaniment to medical appointments. Additional initiatives included nutrition workshops, a community cookbook, and a solidarity-based medication bank. Participants also highlighted strengthened relationships within and between community members and the need for sustainable coordination mechanisms. This study demonstrates the potential of participatory co-creation to generate locally grounded strategies that strengthen social support for chronic disease management. Community governance structures and collective support mechanisms are fundamental in health promotion efforts addressing chronic diseases. Health promotion strategies should integrate community knowledge and participation to enhance their relevance and sustainability.

Contribution to Health Promotion

- This study demonstrates how participatory research can be used to co-create locally relevant strategies to strengthen social support for type 2 diabetes management.
- Community members prioritized collective organizations and mutual aid mechanisms, such as community health committees and accompaniment systems, over purely educational interventions.
- Integrating community knowledge into health promotion design may improve the relevance, ownership, and sustainability of interventions addressing chronic diseases.

## Introduction

Type 2 diabetes mellitus (T2DM) and other chronic diseases place a significant burden on individuals and communities, impacting quality of life and overall well-being (Ledón Llanes 2012, Domínguez Mon 2017). Effectively managing these conditions goes beyond clinical intervention and requires strengthening the social support networks that facilitate self-management (Gallant 2003). Communities are vital assets in this process, playing a key role in health promotion and creating the supportive environments essential for chronic disease management (Willison 2013, Rendón Cazales *et al*. 2024).

Social interactions with communities foster a supportive atmosphere that helps individuals face the challenges of living with T2DM and other chronic diseases (Higa and Davidson 2017). Peer support has proven to be a crucial component in both the prevention and management of chronic diseases, offering individuals with shared experiences a platform to exchange resources and coping strategies, with significant improvements in health outcomes (Funnell 2010, Cartas-Fuentevilla *et al*. 2011, Fisher *et al*. 2015, Warshaw and Edelman 2019, Burgess *et al*. 2022).

Given the critical role of communities in supporting self-management, there is a growing recognition of the value of participatory methods that emphasize co-creation (Agnello *et al*. 2025). By fostering active collaboration among diverse stakeholders, co-creation facilitates the development of health solutions that are both effective and contextually relevant (Plumb *et al*. 2012, Higa and Davidson 2017, Johnson *et al*. 2019, Due-Christensen *et al*. 2021, Song *et al*. 2021, van Eeghen *et al*. 2022). This approach ensures sustainable and culturally relevant interventions, which promotes a sense of shared responsibility among community members (DeVito Dabbs *et al*. 2013). Participatory research has emerged as a particularly effective strategy for chronic disease management, integrating the perspectives of relevant stakeholders to develop interventions tailored to local realities (Leal 2009, Thiollent 2011, Willison 2013, Stone *et al*. 2024). In the specific context of T2DM, a recent systematic review confirms that participatory interventions produce meaningful improvements in glycemic control, particularly when sustained over 6 months and delivered through peer or community health worker facilitation (Ko and Song 2026).

A community-based co-creation approach may therefore be crucial for improving health outcomes in communities facing social vulnerabilities (Lomeli and Rappaport 2018, Gafari *et al*. 2024). Beyond enhancing T2DM self-management, such approaches can strengthen the community’s collective capacity to address chronic health conditions, ensuring that resources are used efficiently and that solutions remain sustainable over time.

This study aims to co-create proposals for improving T2DM management by strengthening social support networks in a community experiencing structural barriers to health and well-being. By engaging community members and stakeholders, we seek to collaboratively identify contextually relevant interventions that empower individuals to take an active role in managing their health. This paper presents a methodological case study documenting the design, implementation, and evaluation of this co-creation process and the transferable insights it generates for participatory health research in low-resource settings.

## Methods

### Theoretical and conceptual framework

Our work is grounded in the participatory research paradigm, which challenges traditional unidirectional models of knowledge production by positioning communities as active co-producers of evidence rather than passive subjects of investigation (Cornwall and Jewkes 1995). This paradigm emphasizes collaborative relationships between academic and community partners, guided by principles of co-learning, mutual benefit, and long-term commitment. It also seeks to democratize knowledge production by valuing community-generated knowledge alongside academic expertise and incorporating community theories, practices, and priorities into the research process.

Within this paradigm, the study draws on sociopraxis principles (Rodriguez-Villasante 2006) as an operational framework. Sociopraxis integrates diverse forms of knowledge through structured participatory processes, facilitating the collective analysis of local realities and supporting the development of culturally relevant and context-sensitive intervention proposals tailored to community needs and resources (López-Sánchez *et al*. 2018, van Eeghen *et al*. 2022).

Co-creation represents the methodological expression of this framework. Rather than a synonym for participation, co-creation refers to iterative cycles of problem identification, solution generation, and collective prioritization in which community members hold meaningful decision-making power (Agnello *et al*. 2025). This approach moves beyond consultation toward shared agency among community members, organizations, and academic partners, ensuring that proposed strategies reflect jointly defined priorities and locally grounded knowledge.

### Study design

This study is part of the CEAD project—Contextualizing Evidence for Action on Diabetes in low-resource Settings: A Mixed-methods case study in Quito and Esmeraldas, Ecuador. From March to September 2024, we conducted a co-creation process in southern Quito involving multiple stakeholders.

Participants in the process included the following:

- The Institute of Public Health (IPH) at the Pontificia Universidad Católica del Ecuador (PUCE).
- “Eloy Alfaro” District Administration—local government authorities.
- Representatives of two citizen organizations: “*Renovación Dorada*” and “*Reino de Quito*.”
- Community members.

### Setting and participants

The technical team involved researchers (four women and one man) from the CEAD project at the University Miguel Hernández in Spain. We established initial community contact through the IPH’s Territorial Health Program (THP), led by the IPH at PUCE in collaboration with Creighton University. The THP aims at strengthening community and academic capacities through service learning, socially relevant research, and contextualized education, advancing community health initiatives to empower local populations and improve health outcomes in southern Quito.

**Figure 1:**
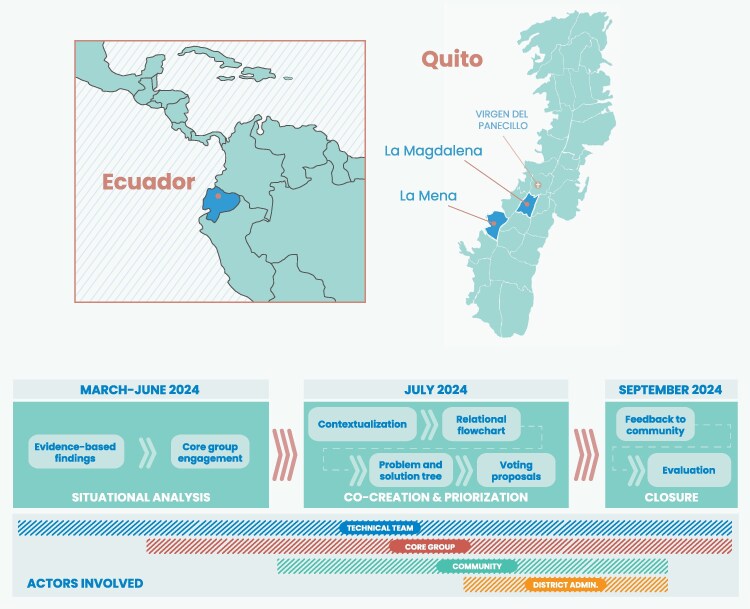
Location and chronogram of the co-creation process and actors involved.

THP researchers recommended two neighborhoods for their well-established community networks and experience in collaborative health initiatives. On one hand, “*La Magdalena*” (37 000 inhabitants in 2019) hosts the “*Renovación Dorada*” association (∼108 participants), active in vaccination campaigns, noncommunicable disease prevention programs, and healthy aging initiatives. On the other hand, “*La Mena*” (53 600 inhabitants) is home to an organization called “*Reino de Quito*” (∼4800 participants), recognized for hosting one of the most active food gardens in the south of Quito (Fig. 1).

### Procedures

The co-creation process was structured in sequential phases, as shown in Fig. 1 along with the timeline and actors involved.

#### Situational analysis

##### Evidence-based findings on T2DM management barriers

To inform the co-creation process, the technical team (all authors) conducted a preliminary situational analysis using both qualitative and quantitative data from the CEAD project. This step aimed to identify key barriers to diabetes management and provide an evidence-informed starting point for stakeholder discussion.

The qualitative component included semi-structured interviews with 16 individuals with diabetes and four healthcare professionals conducted between May and June 2023, exploring experiences of diabetes management and the role of social support. All interviews were audio-recorded with the participants’ permission and transcribed verbatim.

Rather than constituting a standalone qualitative study, the analysis functioned as an action-oriented evidence synthesis designed to guide the participatory process. To achieve this, two members of the technical team (MP-G and MJS-R) independently performed an inductive thematic analysis using ATLAS.ti, with the focus on identifying recurring themes related to perceived barriers and support mechanisms. The analysis involved an iterative reading of the transcripts to extract key practical insights and active discussion to reach a consensus on the key themes. These themes were further discussed within the wider research team to improve analytical coherence and reflexivity. Relevant illustrative quotes were translated from Spanish to English, ensuring the original meaning and context were preserved.

Additionally, quantitative data from a cross-sectional survey involving 570 patients with type 2 diabetes living in southern Quito were incorporated to contextualize and complement the qualitative findings and to strengthen the evidence base presented during the participatory process.

To bridge the gap between academic data and community action, the technical team synthesized and articulated the findings using plain language into 15 “situational analysis reports” briefs that reflect the challenges faced in diabetes management, with particular attention to the role of social support (original reports available at https://zenodo.org/records/14844218). The briefs followed a standardized structure containing: (i) a narrative summary of the problem, (ii) representative qualitative quotes, and (iii) descriptive quantitative metrics to back up the situation. The briefs were used to stimulate dialogue, triangulate experiential knowledge, and support collective prioritization during the co-creation activities. This approach aligns with the methodological objective of the study, which focuses on analyzing the participatory process while integrating empirical evidence to inform deliberation.

##### Core group engagement

To ensure shared ownership of the participatory process, one member of the technical team (SM-G) met with representatives from the IPH, “*Renovación Dorada*,” and “*Reino de Quito*” to present the project’s goals and invite them to form a core group (CG). The situational analysis reports were then presented to all three groups, encouraging individual reflection on the topic and setting the subsequent phases of the co-creation process.

Each group proposed a descriptive title for the challenge and performed a cultural adaptation of the text, ensuring that language and concepts were clear. This step sought to translate technical evidence into accessible language, facilitating meaningful engagement in the next phases of the co-creation process.

The CG comprised seven individuals (two women from “*Renovación Dorada*,” three men from “*Reino de Quito*,” and two representatives from IPH, one woman and one man). The aims of the CG were to coordinate and facilitate participatory workshops, maximizing participation and contributing to communication of results within the broader community. This participatory structure aimed at supporting shared leadership and redistributed facilitation responsibilities beyond the technical team.

#### Co-creation process and prioritization

##### Contextualization

We held a first CG meeting to introduce the participants and organize the co-creation process. This meeting involved capacity strengthening on workshop facilitation with collaborative contextualization of the situational analysis:

###### CG workshop facilitation training

The CG received training in participatory techniques and communication skills to support the facilitation of co-creation workshops, guided by members from the technical team (MP-G and MJS-R). For organizational purposes, a WhatsApp group was created to support ongoing coordination between the technical team and the CG, and a calendar of activities was jointly defined according to community availability. This preparatory phase aimed to enhance local facilitation capacity and promote continuity of engagement beyond individual workshops.

###### CG contextualization of evidence-based findings

The CG reviewed each situational analysis report on diabetes management challenges and collaboratively selected clear and comprehensible titles for each challenge. This step aimed at ensuring clarity and relevance during the following community discussions. In addition, the group co-developed a brief description for each challenge to serve as a discussion prompt and facilitate a shared understanding of the issues, helping to reduce interpretative ambiguity and support more equitable participation across community members (see Supplementary file: SF_Tables and figures, Supplementary Figure a).

##### Community co-creation workshop I: relational flowchart

In the first community workshop, we developed a relational flowchart where each challenge from the situational report was presented on a large poster board. Facilitators read the description from the situation analysis report and addressed participants’ questions. Participants were then asked two key questions: (i) to what extent can the community influence the challenge (ranging from depending on the community to being within their influence or beyond their scope), and (ii) which actor is most responsible for the challenge (individual, family/community, or institutions). Challenges were placed within a matrix to visually categorize these dimensions, providing a visual representation of perceived community influence and responsibility.

**Figure 2:**
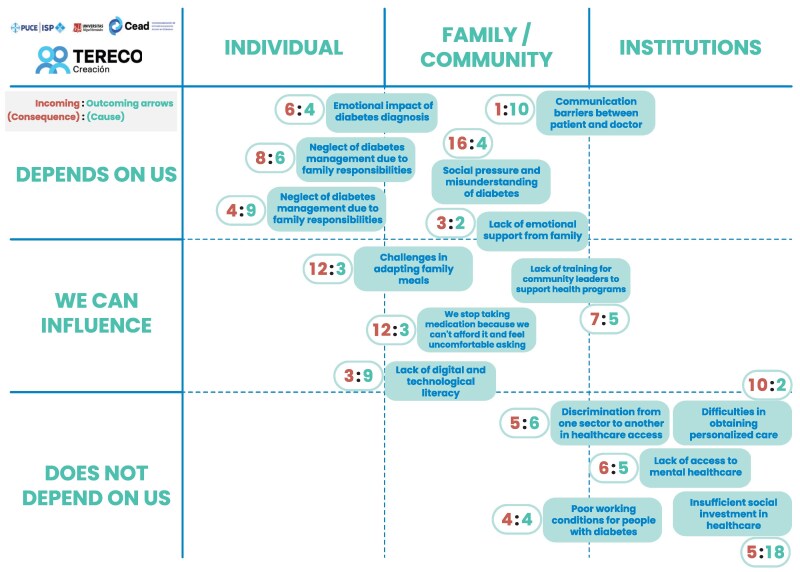
Relational flowchart of diabetes management challenges according to community influence and responsibility.

Participants subsequently mapped relationships between challenges by identifying causal links, represented through directional arrows indicating causes (as outgoing arrows) and consequences (as incoming arrows). This collective mapping exercise aimed to understand the interconnectedness of challenges and support shared understanding of the structural and social determinants influencing diabetes management (Fig. 2).

Following the workshop, the technical team quantified the mapped relationships to identify core challenges. These results informed the development of a “problem and solution tree” for the next phase (see Supplementary file: SF_Flowchart systematization). This analytical step transformed participatory outputs into structured inputs for subsequent deliberation while maintaining methodological transparency in the transition from discussion to synthesis.

##### Community co-creation workshop II: problem and solution tree

The second workshop focused on generating potential solutions to the identified challenges, guided by the question “What can we do to address this problem from within the community?” Participants suggested multiple actions for each challenge, which were collected on Post-it notes and linked to the corresponding items in the problem tree.

A member of the technical team took detailed notes during the discussion to support the following systematization. After the workshop, the technical team grouped similar ideas across both co-creation workshops and shared the final list with the CG for validation and feedback. This process resulted in a non-ranked list of intervention proposals, which formed the basis for the prioritization phase.

##### Prioritization event: voting for proposals

We organized a community event open to the public with the District Administration (DA), which provided logistical and communication support, including infrastructure (setting up a tent, tables, and sound system) and dissemination of the event. For this purpose, we designed an invitation flyer with the CG and distributed it through patient groups and community networks (see Supplementary file: SF_Tables and figures, Supplementary Figure a).

The event location was selected through a joint decision-making process involving the CG and DA following a site visit to potential venues. The final site, “*La Magdalena*” park (intersection of Av. Jacinto Collahuazo and Av. Rodrigo de Chávez), was chosen for its neutrality (situated between both neighborhoods), safety, and accessible environment. This collective selection process aimed to minimize spatial bias and enhance inclusivity in participation.

The prioritization event aimed to generate a collectively ranked list of proposals based on participants’ perceived relevance for strengthening social support strategies in diabetes management. The voting format and scoring system were agreed with the CG prior to the event, including the use of adhesive stickers, a 5–1-point scale, and a total allocation of 15 points per participant. Using a weighted voting method, each participant distributed adhesive stickers with different values (yellow: 5 points; pink: 4 points; green: 3 points; orange: 2 points; red: 1 point), up to a total of 15 points. This procedure was informed by participatory and patient-oriented priority-setting approaches, including dot-voting and point-allocation methods, and by social participation frameworks that emphasize tailoring participatory tools to the social reality, available time, place, resources, and community expectations (Francés *et al*. 2016, Santana *et al*. 2020, Ordunez *et al*. 2022, Tariq *et al*. 2023).

The 15-point allocation was defined in consensus with the CG to balance expressive capacity with procedure simplicity. The 5 to 1 scale allowed participants to express different degrees of preference without requiring a full individual ranking of all proposals. Alternative prioritization approaches were discussed but not adopted, as the selected method was considered more inclusive, allowing participants to engage at their own pace and move freely during the activity. This choice is consistent with priority-setting literature emphasizing that methods should be selected according to the context, time, and resource constraints of the process (Bryant *et al*. 2014).

Prior to the event and together with the CG, we defined a series of correction factors for the voting system, which doubled the votes if the proposal (i) could be implemented by the community without external support, (ii) contributed to strengthening social support networks, and (iii) addressed root causes identified in the problem tree. These correction factors were guided by the study’s core objectives of promoting community ownership, strengthening social support, and addressing structural root causes of poor diabetes management. They were not intended to replace participants’ preferences but to make explicit the degree of alignment between each proposal and the objectives of the process. Thus, the final score combined community preference, captured through the weighted vote, with feasibility and strategic alignment with the participatory diagnosis. This is consistent with participatory and health priority-setting literature, which recommends using explicit, transparent, and context-sensitive criteria when combining community priorities with feasibility, relevance, and action-oriented criteria (Salihu *et al*. 2015, Tong *et al*. 2019). Final scores were calculated, and proposals were ranked to create the community’s prioritized list. The resulting ranking should therefore be interpreted as an adjusted participatory prioritization.

#### Closure

##### Results devolution to the community

The CG first reviewed and validated the outcomes, providing feedback on the interpretation of the prioritization outcomes and reflecting on the overall co-creation process. To facilitate dissemination, a community-friendly, visually trifold brochure was developed summarizing the prioritized proposals (Supplementary file: SF_Tables and figures, Supplementary Figure b).

The final results were then presented by the CG to the broader community through dissemination activities using clear, nontechnical language and visual formats to ensure accessibility and comprehension.

##### Evaluation

Following dissemination, a multilayered evaluation of the process was conducted. Two CG members from IPH facilitated separate evaluations with representatives from “*Renovación Dorada*” and “*Reino de Quito*.” Additionally, a researcher from PUCE, external to the process, carried out the same evaluation with the two CG members from IPH.

The evaluation used a tetralemma matrix (done/not done; like/dislike; see Supplementary file: SF_Tables and figures, Supplementary Figure c) to structure reflection on the process. We aimed to encourage in-depth discussions about the implemented strategies, highlighting positive, negative, and any overlooked aspects of the process.

All discussions were documented through this matrix and supplemented with field notes. Subsequently, one member of the research team (EC-R) synthesized the three independent evaluations to provide an integrated assessment of the co-creation process.

### Ethical approval and informed consent

The participatory research is included in the CEAD protocol, which has been reviewed and approved by the ethical review board at PUCE (reference 2019-27-MB). Furthermore, as this study was developed within a European Research Council-funded program implemented in a low- and middle-income country, the project was subject to regular ethical monitoring and supervision by the European Research Council Executive Agency and an appointed external ethical advisor. Participants provided written informed consent after being fully informed about the study purpose, procedures, potential risks, benefits, and the right to withdraw at any time without any negative consequence. No individual sociodemographic data were recorded during the co-creation phase. All quantitative and qualitative data collected within the scope of the project were strictly anonymized and managed in compliance with established data protection protocols.

## Results

### Situational analysis

Following the initial workshop with the CG, titles for the challenges were collaboratively refined and, where necessary, reformulated (Table 1).

**Table 1:** List of key type 2 diabetes management challenges labeled by the core group.

Scope	Challenges in T2DM^a^
Social and community context	Lack of emotional support from family Social pressure and misunderstanding of diabetes Neglect of diabetes management due to family responsibilities Challenges in adapting family meals
Economic stability	We stop taking medication because we can't afford it and feel uncomfortable asking Poor working conditions for people with diabetes
Education	Limited knowledge about the disease and its management Lack of digital and technological literacy Lack of training for community leaders to support health programs
Healthcare system	Communication barriers between patient and doctor Insufficient social investment in healthcare Difficulties in obtaining personalized care Discrimination from one sector to another in healthcare access

^a^Full description of each challenge is available in Supplementary file: SF_Tables and figures, Supplementary Table a.

### Participatory co-creation process and prioritization

#### Relational flowchart

A total of 124 participants engaged in the relational flowchart workshops across both communities. On July 10, 67 participants attended the workshop in “*Reino de Quito*,” while 57 participated in “*Renovación Dorada*” on July 11. Given the high participation, participants were divided into three groups to facilitate discussion. Guided by the CG and the technical team, the community classified challenges (Table 1) and mapped causal relationships on a flowchart (Fig. 2).

**Figure 3:**
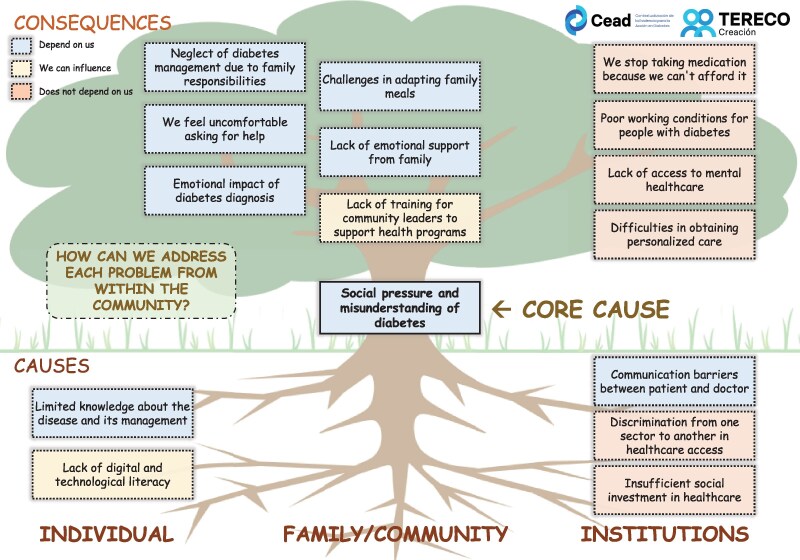
Problem tree of the selected core challenge, showing causes, consequences, and participant-defined levels of agency.

All the technical team analyzed the six resulting flowcharts in Excel, consolidating information by counting entry and exit arrows for each challenge (see Supplementary file: SF_Flowchart systematization). The most significant challenges were identified as “lack of social investment in health” (23 arrows, 18 as a cause) and “social pressure and misunderstanding of diabetes” (20 arrows, 16 as a consequence). We selected the latter as the core problem due to its higher perceived influence, as determined by voting. This choice guided the structure of the problem tree narrative, with challenges identified as causes positioned below the trunk and as consequences in the branches (Fig. 3). The position of the challenge was based on the responsible agent, with different colors indicating levels of agency as defined by participants: red (out of reach), yellow (we can influence), and blue (within our control).

#### Problem and solution tree

On July 15, 18 participants from “*Renovación Dorada*” attended the workshop, followed by 10 participants from “*Reino de Quito*” on July 17. To enhance engagement and ensure effective participation, we held a single group session in each community, resulting in two independent solution trees. Starting with the blue challenges (within our control), we facilitated discussions to gather proposals linked to each challenge, writing in Post-it the different ideas. The technical team then used these ideas, along with field notes, to summarize the different proposals and create a draft list that was later confirmed by the core group.

### Prioritization event

The prioritization event took place on July 24th at Parque La Magdalena, with a total of 70 participants voting for their preferred options (see Supplementary file: SF_Tables and figures, Supplementary Figure d). During the event, both groups showcased activities aligned with their interests, including an ecological composting workshop by the “*Reino de Quito*” committee and a traditional dance performance by the “*Renovación Dorada*” dance group. These activities enriched the event, promoting knowledge-sharing and strengthening intergroup relationships. Moreover, members of the broader community who attended got to learn about the groups and were encouraged to get involved, promoting greater engagement and inclusivity in the neighborhoods.

#### Final list of prioritized proposals

As a result of the systematization (see Supplementary file: SF_Tables and figures, Supplementary Table b), a final list of prioritized proposals was elaborated and grouped around scopes while respecting the points obtained in each (Table 2). The most voted proposal, the creation of a community health commission, received 1122 points. The results were shared in community meetings on September 11th in “*Reino de Quito*” and September 12th in “*Renovación Dorada*.”

**Table 2:** List of co-created proposals grouped by scope.

Scope	Order (points)	Community co-created proposals
Social support	1st (1122)	Creation of a community health commission with activities such as identifying people with limited family support, raising family awareness of the needs of people with diabetes, providing accompaniment to medical appointments, assessing home needs, and organizing fundraising activities.
	7th (200)	Request for psychological care professionals at the health center to hold regular (bi-weekly) conversations with chronic disease patient clubs.
	14th (92)	Organization of conversations to share difficulties in managing diabetes, creating a safe space for advice and emotional support within the group.
	15th (66)	Organization of social theater workshops to strengthen community bonds and perform skits depicting the daily lives of people with diabetes, raising awareness of their needs.
Promotion of healthy eating	2nd (936)	Organization of nutritional cooking workshops that include affordable recipes and preparation of traditional, healthy dishes.
	3rd (426)	Creation of a recipe book with traditional and healthy dishes to share nutritious recipes, including those suitable for conditions like diabetes and hypertension.
	6th (366)	Healthy cooking contest with traditional recipes to revive dishes like “*chapo*,” “*morocho*,” and “*chuchuca*,” promoting their preparation.
Social support for resources	4th (398)	Creation of a nonprofit medication bank to provide medicine to group members in need.
	12th (116)	Economic sponsorship within the patient club or neighborhood organization: financial support from a “godparent” for those in need, covering medication costs, group fees, etc.
Encouragement of food gardens	5th (366)	Organization of intergenerational collaboration activities, where youth learn composting, planting, harvesting, and healthy recipes in exchange for helping older adults with social media skills.
	13th (96)	Promotion of urban gardening among youth with school visits and the reintroduction of natural sciences in schools to increase youth knowledge on ecological and healthy eating.
Information dissemination and awareness	8th (192)	Collection of community signatures to request health fairs and medical brigades to provide care and information on chronic disease prevention and management.
	10th (147)	Request to the health center for talks and workshops on diabetes within the community to improve understanding of chronic diseases.
	11th (128)	Dissemination of information on diabetes prevention and care through neighborhood WhatsApp groups and informative posters in community centers and common areas to reach people without social media.
	16th (40)	Training community health agents to spread information on diabetes and other preventable diseases, including outreach to families of people with these chronic illnesses.
Community empowerment	9th (168)	Collaboration between neighborhood organizations with regularly scheduled joint activities, enhancing the training of community leaders. Example: Handicraft workshops in “*Renovación Dorada*” and cooking workshops in “*Reino de Quito*.”

**Figure 4:**
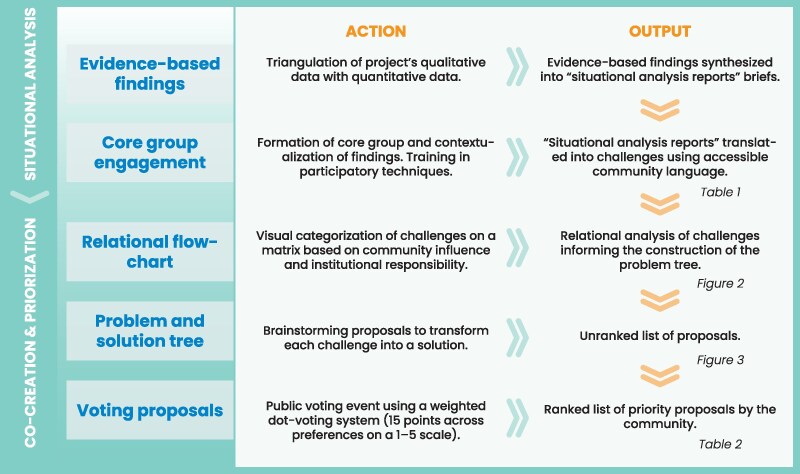
Overview of the research co-creation process, illustrating the alignment between methodological actions, outputs, and embedded manuscript components.

The comprehensive participatory methodology employed in this study and sequential outputs are visualized in Fig. 4. This flowchart outlines the connection between data triangulation, core group engagement, and the sequential co-creation steps used to surface and rank community proposals.

### Evaluation

In the evaluation, the CG highlighted positive aspects of the process. Notably, they emphasized the relationships built both within the CG and among the community members who participated in the work carried out. The process allowed mutual learning and collaboration between both associations. For example, the “*Reino de Quito*” committee, with extensive experience in urban garden management, offered to share its knowledge with members of “*Renovación Dorada*,” who were very interested in understanding the model of urban gardens and establishing synergies that would allow their participants to access part of the harvest at affordable prices, as well as participating in the workshops on healthy eating. In addition, the knowledge acquired throughout the process was highly valued by both the CG and the participating community members. Issues such as “*more awareness about diabetes and hypertension*” or “*exchanging ideas*” were noted on the tetralemma matrix in the “like” category.

The community particularly appreciated the opportunity to connect with the University. However, concerns were expressed regarding the continuity and implementation of the proposals, as well as a lack of coordination with other strategic programs the University was developing in the area. “Having mechanisms for continuity of the proposals and implementation” and “having more meetings and continuing things” were noted in the “dislike” category. To address this, the CG proposed better coordination through co-design between projects operating in the same territory, alongside long-term, sustainable planning to ensure the proposals’ lasting impact.

## Discussion

### Key findings

This study describes a structured co-creation process aimed at generating and prioritizing community-driven proposals to improve T2DM management by strengthening social support networks in southern Quito, Ecuador. Its contribution goes beyond applying participatory research principles in a low-resource, urban Latin American setting. The findings show that communities frame chronic disease management as a social and structural process, rather than an individual behavior problem, and the co-creation process itself generates cohesion and collective agency that communities can draw on for local health action. The process generated proposals addressing multidimensional barriers, including social, psychological, economic, and structural factors. The highest-ranked proposal was the creation of a community health commission, reflecting the recognition of social isolation and limited family support as barriers to effective diabetes care (Rosland *et al*. 2010). Through activities such as accompanying patients to medical visits and conducting home assessments, this proposal underscores the centrality of collective structures and community organization in addressing chronic disease challenges (Plumb *et al*. 2012).

The co-created proposals extended beyond individual behavior change, emphasizing peer support, culturally relevant dietary practices, psychological support, and community-based solidarity mechanisms. These priorities indicate that participants conceptualized diabetes management as a social process shaped by relational and structural determinants, reflecting a holistic understanding of health. The emphasis on community-led governance and advocacy also supports the idea of shifting toward participatory approaches to health decision-making, where communities seek to mobilize resources and influence local health agendas.

In addition to generating intervention proposals, the co-creation process also strengthened relationships among community members and between community organizations and academic partners. These relational outcomes highlight the potential of participatory approaches to foster social cohesion and collective agency, which are themselves important determinants of health promotion.

### Comparison with literature

This experience contributes to a still limited body of literature on the application of co-creation approaches to chronic disease management in a low-resource setting in Latin America (Hoon Chuah *et al*. 2018). Although participatory research has a long tradition in the region, it has historically focused on infectious disease or malnutrition, following the epidemiological priorities of the region (Mercado-Martínez Robles-Silva and Jiménez-Domínguez 2018). Consequently, co-creation applied to T2DM remains underdeveloped despite the growing burden of noncommunicable diseases.

Our findings are consistent with a systematic review identifying only 22 co-creation studies in public health conducted across low- and middle-income countries, four in Latin America (Longworth *et al*. 2024). Although our study did not reach the implementation phase of the co-created proposals, we identified similar challenges and facilitating factors during the co-creation process itself. Structural and financial constraints, together with concerns about sustainability, emerged as key challenges. In contrast, sustained community involvement, trust-building, and adaptation to the local context facilitated participation and engagement throughout the process. These process dynamics are reflected in the content of the co-created proposals, which translate relational and contextual priorities into concrete community-oriented strategies for diabetes management.

The different proposals prioritized by the community align with existing evidence on the importance of social support in diabetes management. Community-based initiatives such as peer accompaniment, home visits, peer-led conversations, and psychological support reflect an understanding that living with a chronic condition demands social support, which is not just a complement to medical care but a key determinant of overall health (Kalra Jena and Yeravdekar 2018). The emphasis on culturally relevant and affordable dietary changes echoes public health literature highlighting the importance of integrating local knowledge and traditions into health promotion strategies to enhance long-term adherence and effectiveness (Campos Navarrete and Zohar 2021). Participants also suggested more innovative approaches, such as social theater workshops, which have been shown to foster empathy and strengthen community bonds (Killick 2020, Viola *et al*. 2024).

Economic barriers were a recurring concern, reflected in proposals such as a nonprofit medication-sharing bank and financial sponsorship initiatives within the health-based group. These illustrate a model of community-driven solidarity, where local networks mobilize internal resources to overcome critical healthcare gaps (Cartas-Fuentevilla *et al*. 2011). Finally, proposals related to local advocacy and community leadership point to a broader shift in how communities conceptualize their role in health as active agents capable of mobilizing resources, influencing local policy, and co-developing solutions for health promotion (Fawcett *et al*. 1995, Dushkova and Ivlieva 2024).

### Strengths and limitations

This study has notable strengths. The integration of multiple knowledge sources, combining a prior evidence-based situational analysis with community-based feedback, facilitated informed participation while maintaining community ownership through explicit contextualization and validation by the CG. Although the situational analysis was not directly generated by participants during the process, it drew from 4 years of extensive mixed-methods research, integrating quantitative and qualitative data from patients, caregivers, and healthcare professionals in the area, which provided a rigorous empirical foundation for community deliberation without imposing external priorities.

The diversity among participating communities also enriched the process. “*Renovación Dorada*,” already familiar with T2DM, readily engaged with the disease management challenges, while “*Reino de Quito*,” though less familiar with the condition clinically, brought strong understanding of community as a structural factor in health promotion. The involvement of diverse stakeholders supported shared ownership and increased the relevance of the proposed strategies.

A further strength was the continuous community lens embedded throughout the participatory activities: by systematically framing problems and solutions through collective rather than individual matrices, the process enabled shared discourses and structural reframing that would be unlikely to emerge through more conventional research methodologies.

Regarding limitations, this study focused on the co-creation and prioritization of proposals without evaluating their implementation or impact. This was not an unforeseen gap but a deliberate methodological scope: the process was designed as part of the broader THP already underway in collaboration with the university, with the explicit aim of generating a prioritized roadmap that communities could use internally. Both community groups were aware of this from the outset, and managing expectations about the boundaries of the study was an integral part of the process design. Nevertheless, the capacity for continuity beyond the research period remains a recognized challenge in participatory health research, and future studies should address implementation pathways more explicitly from the start (Agnello *et al*. 2025).

Another limitation for replicability is that both selected communities had strong preexisting organizational structures and prior academic engagement. In contexts without these preexisting favorable conditions, more time may be needed to build trust and foster collaboration. While this strong foundation enabled a more dynamic process, it also required careful consideration to avoid overburdening community members. For this reason, the duration of the process was intentionally limited, which may have influenced community engagement.

Although CG members were invited to all workshops to minimize intergroup power dynamics and encourage a shared perspective, time constraints prevented some representatives from “*Reino de Quito*” from attending workshops in “*Renovación Dorada*.” This may have reduced the collective vision that a fully integrated cross-community process could have generated.

An important dynamic also emerged around facilitation leadership. The CG explicitly requested greater involvement from the technical team during workshops, recognizing that participants might engage more readily with an academic institution visibly co-leading the sessions. As a result, the technical team assumed a more active co-facilitation role than originally planned. While we do not interpret this as a failure of the participatory process, it raises important considerations. The presence of academic facilitators may have subtly shaped the deliberative space, influencing which voices felt confident to contribute, which proposals were articulated most clearly, and how collective priorities were framed. This is an important challenge in shifting toward more horizontal, community-driven health initiatives in contexts where vertical structures remain dominant (Popay *et al*. 2021). For future participatory research, this highlights the need to invest in sustained, iterative community facilitation capacity as a long-term process of redistributing methodological agency. The degree of horizontal participation should be evaluated explicitly as a process outcome, rather than assuming it from the methodology’s design. We also recommend addressing facilitation expectations and power-sharing arrangements explicitly at the outset of any similar process.

Finally, although the weighted voting system was co-defined with the CG, it proved unsatisfactory in practice. Participants wished to vote for all proposals but were constrained by the punctuation and maximum number of stickers available per person, leading some to place lower-value stickers in the highest-value category once their top-scoring stickers ran out. To ensure robustness, we recalculated results using both numerical and color-based scoring systems; although four proposals shifted slightly in position, the overall ranking remained unchanged. We also noted a potential positional bias, as proposals listed first may have received disproportionate attention. Together, these issues suggest that the sticker allocation constrained participants’ ability to fully express their preferences. Future studies should pilot alternative prioritization methods and adjust according to community wishes and context.

### Practice implications

The proposals emerging from this co-creation process highlight the transformative potential of community-driven approaches to diabetes management. As emphasized by Popay *et al*., it is crucial to recognize communities’ ability to address health challenges (power within) and their capacity to collaborate with others (power with) (Popay *et al*. 2021). Health authorities should engage with communities to enhance their collective action (power to) to address diabetes comprehensively. In contexts like southern Quito, where an underfunded, fragmented, and weakened healthcare system exacerbates health inequalities, integrating grassroots solutions into policy frameworks is essential to bridge systemic gaps and drive structural transformations that strengthen chronic disease care at both local and institutional levels.

While the specific proposals generated in this study are not intended to be generalizable, as their value lies precisely in their contextual relevance, the methodology itself demonstrates transferable potential. Furthermore, the community’s collectivist ideas for T2DM management extend beyond this particular process, serving as a practical starting point for discussion and innovation in other settings facing similar challenges. Participatory co-creation processes, even under time constraints, can strengthen community cohesion, foster collective discourse, and surface structural determinants of health that conventional approaches tend to overlook.

This study also points to two specific policy priorities. First, integrating evidence-informed situational analyses into participatory processes can support community deliberation while preserving local ownership, offering a replicable model for chronic disease research in low-resource settings. Second, sustained investment in community facilitation capacity is essential: the hierarchical dynamics observed here are not a community deficit but a structural consequence of how health research and care have historically been organized in Latin American contexts, where paternalistic relationships persist (Elío-Calvo 2020). Institutions engaging in participatory research must commit to longer, iterative capacity-building processes that genuinely redistribute methodological agency to community partners (Wallerstein *et al*. 2019).

Finally, this study underscores the urgency of bridging the gap between co-creation and implementation. Sustained collaboration between communities, academic institutions, and health authorities is necessary to translate prioritized proposals into actionable interventions and to ensure that participatory processes produce lasting health impact rather than well-documented but unimplemented priorities.

## Conclusion

This study demonstrates that structured co-creation processes can generate contextually relevant, community-endorsed priorities for chronic disease management in low-resource urban settings. By systematically framing health challenges through a collective rather than individual lens, the participatory process enables communities to transcend dominant narratives of personal responsibility and focus on structural, solidarity-based responses to chronic diseases.

While the specific proposals generated here are not intended to be replicated elsewhere, we highlight the methodological approach itself: the integration of prior evidence into participatory deliberation, the use of structured co-creation tools to foster collective discourse, and the role of universities as trusted bridges between communities and public institutions to build trust and enable long-term impact. Fostering synergies between independent social organizations with different backgrounds is key to promoting cooperation, improving knowledge exchange, and building the relational infrastructure that sustains community-driven health initiatives over time.

In contexts where health systems are fragmented and under-resourced, this approach offers a pathway to strengthen community support networks, reduce health inequities, enhance local capacity, and empower communities to address health challenges.

## Supplementary Material

daag123_Supplementary_Data

## Data Availability

All data generated or analyzed during this study are included in this published article and its supplementary information files. Original “situational analysis reports” are available in 10.5281/zenodo.14844218
