# Supplementary material for: Co-creating community support strategies for chronic disease management: insights from our experience with two community groups in southern Quito, Ecuador
Source: Health Promot Int. 2026 Aug 18;41(4):daag123. doi: 10.1093/heapro/daag123 (PMC13482984; doi:10.1093/heapro/daag123)
Supplement: Supplementary file 1 [file daag123_supplementary_data.zip › SF_Flowchart systematization.docx]

| Flowchart systematization | | | | | | | | | | | | | |
| --- | --- | --- | --- | --- | --- | --- | --- | --- | --- | --- | --- | --- | --- |
|  |  |  |  | **Dimensions** | | **Relationships** | | | | |  |  |  |
|  | RQ:Reino de Quito RD:Renovación Dorada | **Place** | **Group** | **Community influence** | **Responsible actor** | **Incoming (consequence)** | **TOTAL Incoming** | **Outgoing (causes)** | **TOTAL Outgoing** | **Total incoming arrows** consequences | **Total outgoing arrows** causes | **Total number of arrows** | **Positive (consequence) / negative (cause)** |
| **1** | Emotional impact of diabetes diagnosis | RQ | 1 | Depends on us | Individual | 3 | 3 | 0 | 2 | 6 | 4 | 10 | 2 |
|  |  | RQ | 2 | Depends on us | Family-community | 0 |  | 1 |  |  |  |  |  |
|  |  | RQ | 3 | We can influence | Individual | 0 |  | 1 |  |  |  |  |  |
|  |  | RD | 1 | Depends on us | Individual | 1 | 3 | 1 | 2 |  |  |  |  |
|  |  | RD | 2 | Depends on us | Family-community | 1 |  | 0 |  |  |  |  |  |
|  |  | RD | 3 | Depends on us | Individual/Family-community/Institution | 1 |  | 1 |  |  |  |  |  |
| **2** | Limited knowledge about the disease and its management | RQ | 1 | Depends on us | Individual | 1 | 2 | 1 | 4 | 4 | 9 | 13 | -5 |
|  |  | RQ | 2 | We can influence | Institution | 1 |  | 1 |  |  |  |  |  |
|  |  | RQ | 3 | Depends on us | Individual | 0 |  | 2 |  |  |  |  |  |
|  |  | RD | 1 | Depends on us | Family-community | 1 | 2 | 1 | 5 |  |  |  |  |
|  |  | RD | 2 | Depends on us / We can influence | Institution | 1 |  | 0 |  |  |  |  |  |
|  |  | RD | 3 | Depends on us | Individual | 0 |  | 4 |  |  |  |  |  |
| **3** | Communication barriers between patient and doctor | RQ | 1 | Depends on us | Family-community | 0 | 1 | 5 | 7 | 1 | 10 | 11 | -9 |
|  |  | RQ | 2 | Depends on us | Institution | 1 |  | 1 |  |  |  |  |  |
|  |  | RQ | 3 | Depends on us | Individual | 0 |  | 1 |  |  |  |  |  |
|  |  | RD | 1 | Depends on us | Institution | 0 | 0 | 1 | 3 |  |  |  |  |
|  |  | RD | 2 | It does not depend on us | Institution | 0 |  | 1 |  |  |  |  |  |
|  |  | RD | 3 | Depends on us | Individual | 0 |  | 1 |  |  |  |  |  |
| **4** | Lack of emotional support from family | RQ | 1 | Depends on us | Family-community | 0 | 6 | 5 | 8 | 3 | 2 | 5 | 1 |
|  |  | RQ | 2 | Depends on us | Family-community | 4 |  | 1 |  |  |  |  |  |
|  |  | RQ | 3 | It does not depend on us | Family-community | 2 |  | 2 |  |  |  |  |  |
|  |  | RD | 1 | We can influence | Family-community | 0 | 1 | 4 | 11 |  |  |  |  |
|  |  | RD | 2 | Depends on us | Family-community | 0 |  | 4 |  |  |  |  |  |
|  |  | RD | 3 | Depends on us | Family-community | 1 |  | 3 |  |  |  |  |  |
| **5** | Lack of access to mental healthcare | RQ | 1 | It does not depend on us | Institution | 1 | 1 | 2 | 5 | 6 | 5 | 11 | 1 |
|  |  | RQ | 2 | We can influence | Institution | 0 |  | 1 |  |  |  |  |  |
|  |  | RQ | 3 | Depends on us | Institution | 0 |  | 2 |  |  |  |  |  |
|  |  | RD | 1 | It does not depend on us | Institution | 1 | 5 | 0 | 0 |  |  |  |  |
|  |  | RD | 2 | It does not depend on us | Institution | 2 |  | 0 |  |  |  |  |  |
|  |  | RD | 3 | It does not depend on us | Institution | 2 |  | 0 |  |  |  |  |  |
| **6** | Insufficient social investment in healthcare | RQ | 1 | It does not depend on us | Institution | 1 | 3 | 3 | 8 | 5 | 18 | 23 | -13 |
|  |  | RQ | 2 | It does not depend on us | Institution | 1 |  | 1 |  |  |  |  |  |
|  |  | RQ | 3 | Depends on us | Institution | 1 |  | 4 |  |  |  |  |  |
|  |  | RD | 1 | We can influence | Institution | 1 | 2 | 3 | 10 |  |  |  |  |
|  |  | RD | 2 | It does not depend on us | Institution | 0 |  | 5 |  |  |  |  |  |
|  |  | RD | 3 | We can influence | Institution | 1 |  | 2 |  |  |  |  |  |
| **7** | Difficulties in obtaining personalized care | RQ | 1 | We can influence | Institution | 2 | 7 | 1 | 1 | 10 | 2 | 12 | 8 |
|  |  | RQ | 2 | It does not depend on us | Institution | 2 |  | 0 |  |  |  |  |  |
|  |  | RQ | 3 | It does not depend on us | Institution | 3 |  | 0 |  |  |  |  |  |
|  |  | RD | 1 | It does not depend on us | Institution | 2 | 3 | 0 | 1 |  |  |  |  |
|  |  | RD | 2 | Depends on us | Institution | 0 |  | 1 |  |  |  |  |  |
|  |  | RD | 3 | It does not depend on us | Institution | 1 |  | 0 |  |  |  |  |  |
| **8** | Neglect of diabetes management due to family responsibilities | RQ | 1 | Depends on us | Individual | 1 | 3 | 0 | 0 | 7 | 1 | 8 | 6 |
|  |  | RQ | 2 | Depends on us / We can influence | Individual | 1 |  | 0 |  |  |  |  |  |
|  |  | RQ | 3 | Depends on us | Family-community | 1 |  | 0 |  |  |  |  |  |
|  |  | RD | 1 | Depends on us | Family-community | 2 | 4 | 0 | 1 |  |  |  |  |
|  |  | RD | 2 | Depends on us | Family-community | 0 |  | 1 |  |  |  |  |  |
|  |  | RD | 3 | Depends on us | Individual | 2 |  | 0 |  |  |  |  |  |
| **9** | Lack of training for community leaders to support health programs | RQ | 1 | We can influence | Family-community/Institution | 2 | 3 | 1 | 3 | 7 | 5 | 12 | 2 |
|  |  | RQ | 2 | We can influence | Family-community/Institution | 0 |  | 1 |  |  |  |  |  |
|  |  | RQ | 3 | Depends on us | Family-community | 1 |  | 1 |  |  |  |  |  |
|  |  | RD | 1 | We can influence | Family-community/Institution | 2 | 4 | 1 | 2 |  |  |  |  |
|  |  | RD | 2 | It does not depend on us | Institution | 1 |  | 1 |  |  |  |  |  |
|  |  | RD | 3 | It does not depend on us | Institution | 1 |  | 0 |  |  |  |  |  |
| **10** | We stop taking medication because we can't afford it and feel uncomfortable asking | RQ | 1 | Depends on us / We can influence | Family-community/Institution | 2 | 5 | 0 | 2 | 12 | 3 | 15 | 9 |
|  |  | RQ | 2 | Depends on us | Individual/Family-community | 1 |  | 1 |  |  |  |  |  |
|  |  | RQ | 3 | Depends on us | Individual | 2 |  | 1 |  |  |  |  |  |
|  |  | RD | 1 | We can influence | Institution | 3 | 7 | 0 | 1 |  |  |  |  |
|  |  | RD | 2 | Depends on us | Individual/Family-community | 3 |  | 1 |  |  |  |  |  |
|  |  | RD | 3 | It does not depend on us | Family-community/Institution | 1 |  | 0 |  |  |  |  |  |
| **11** | Challenges in adapting family meals | RQ | 1 | Depends on us | Individual/Family-community | 4 | 7 | 0 | 1 | 12 | 3 | 15 | 9 |
|  |  | RQ | 2 | Depends on us | Individual | 2 |  | 1 |  |  |  |  |  |
|  |  | RQ | 3 | Depends on us | Family-community | 1 |  | 0 |  |  |  |  |  |
|  |  | RD | 1 | We can influence | Family-community | 2 | 5 | 0 | 2 |  |  |  |  |
|  |  | RD | 2 | Depends on us | Individual/Family-community | 2 |  | 1 |  |  |  |  |  |
|  |  | RD | 3 | We can influence | Family-community | 1 |  | 1 |  |  |  |  |  |
| **12** | Discrimination from one sector to another in healthcare access | RQ | 1 | It does not depend on us | Institution | 0 | 2 | 2 | 5 | 5 | 6 | 11 | -1 |
|  |  | RQ | 2 | It does not depend on us | Institution | 0 |  | 3 |  |  |  |  |  |
|  |  | RQ | 3 | We can influence | Institution | 2 |  | 0 |  |  |  |  |  |
|  |  | RD | 1 | It does not depend on us | Institution | 0 | 3 | 1 | 1 |  |  |  |  |
|  |  | RD | 2 | It does not depend on us | Institution | 2 |  | 0 |  |  |  |  |  |
|  |  | RD | 3 | We can influence | Family-community/Institution | 1 |  | 0 |  |  |  |  |  |
| **13** | Social pressure and misunderstanding of diabetes | RQ | 1 | It does not depend on us | Individual/Family-community | 6 | 10 | 1 | 3 | 16 | 4 | 20 | 12 |
|  |  | RQ | 2 | Depends on us | Individual | 4 |  | 1 |  |  |  |  |  |
|  |  | RQ | 3 | Depends on us | Individual | 0 |  | 1 |  |  |  |  |  |
|  |  | RD | 1 | Depends on us | Family-community | 3 | 6 | 1 | 1 |  |  |  |  |
|  |  | RD | 2 | Depends on us | Individual | 2 |  | 0 |  |  |  |  |  |
|  |  | RD | 3 | Depends on us | Family-community | 1 |  | 0 |  |  |  |  |  |
| **14** | Lack of digital and technological literacy | RQ | 1 | Depende de nosotros/Podemos influir | Individual | 0 | 1 | 2 | 3 | 3 | 9 | 12 | -6 |
|  |  | RQ | 2 | We can influence | Family-community | 0 |  | 1 |  |  |  |  |  |
|  |  | RQ | 3 | We can influence | Institution | 1 |  | 0 |  |  |  |  |  |
|  |  | RD | 1 | Depends on us | Individual | 1 | 2 | 3 | 6 |  |  |  |  |
|  |  | RD | 2 | Depends on us | Individual | 1 |  | 0 |  |  |  |  |  |
|  |  | RD | 3 | We can influence | Family-community | 0 |  | 3 |  |  |  |  |  |
| **15** | Poor working conditions for people with diabetes | RQ | 1 | It does not depend on us | Institution | 0 | 1 | 2 | 3 | 4 | 4 | 8 | 0 |
|  |  | RQ | 2 | Depends on us | Institution | 0 |  | 1 |  |  |  |  |  |
|  |  | RQ | 3 | We can influence | Institution | 1 |  | 0 |  |  |  |  |  |
|  |  | RD | 1 | It does not depend on us | Institution | 0 | 3 | 1 | 1 |  |  |  |  |
|  |  | RD | 2 | We can influence | Institution | 1 |  | 0 |  |  |  |  |  |
|  |  | RD | 3 | It does not depend on us | Institution | 2 |  | 0 |  |  |  |  |  |
