# Supplementary material for: Co-creating community support strategies for chronic disease management: insights from our experience with two community groups in southern Quito, Ecuador
Source: Health Promot Int. 2026 Aug 18;41(4):daag123. doi: 10.1093/heapro/daag123 (PMC13482984; doi:10.1093/heapro/daag123)
Supplement: Supplementary file 1 [file daag123_supplementary_data.zip › SF_Tables and figures_Figure A.pdf]

TEJIENDO REDES COMUNITARIAS

TERECO Creación

Cead

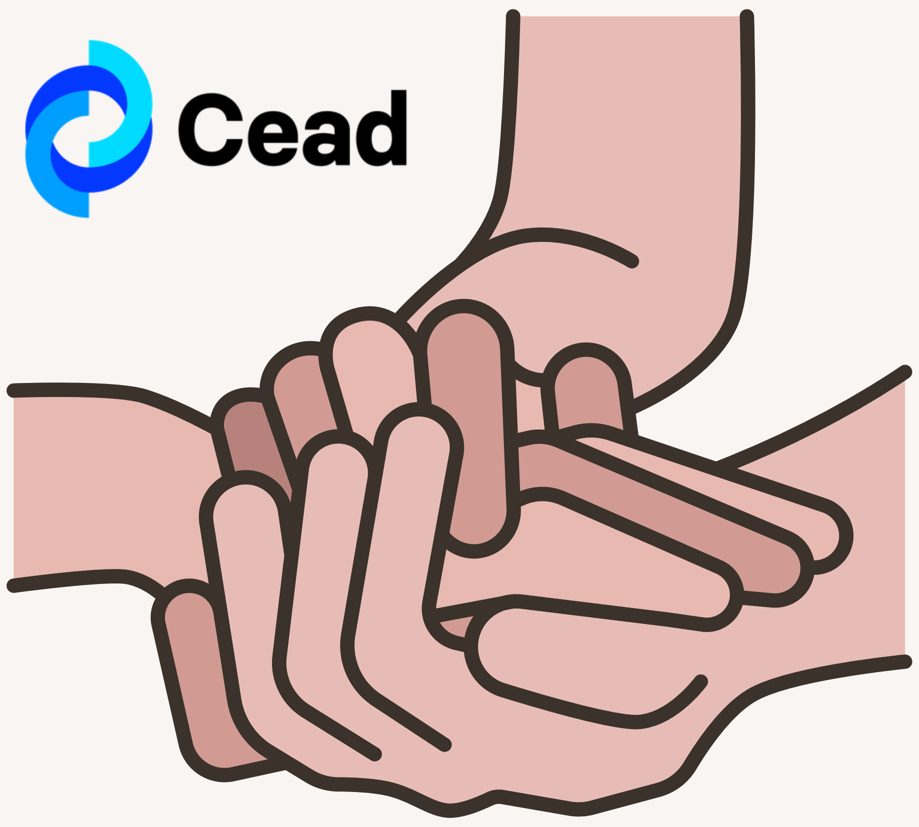

24 DE JULIO 2024

ACCESO LIBRE DE 10 A 13 AM

Parque La Magdalena, frente estación metro (Av. Jacinto Collahuazo y Av. Rodrigo de Chávez) 170111, **Quito**

CASA ABIERTA

¡Votemos entre todos las mejores propuestas para fortalecer las redes de apoyo en su comunidad y mejorar nuestra salud!

LES INVITAMOS A CONOCER ESTRATEGIAS PROPUESTAS POR LA COMUNIDAD PARA TEJER REDES DE APOYO SOCIAL Y SER MÁS SALUDABLES

¡NECESITAMOS DE SU COLABORACIÓN PARA ELEGIR LAS MEJORES IDEAS!

ADEMÁS CONTAREMOS CON MÚSICA Y OTRAS ACTIVIDADES:

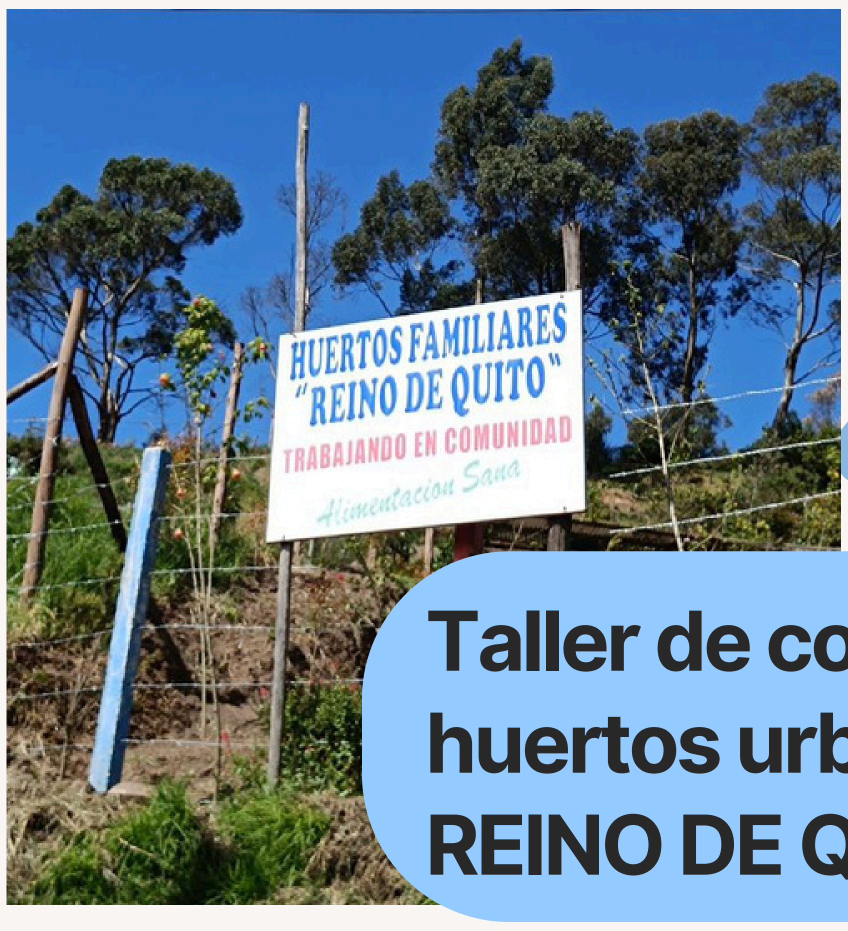

Taller de compostaje huertos urbanos REINO DE QUITO

Actuación grupo de danza RENOVACIÓN DORADA

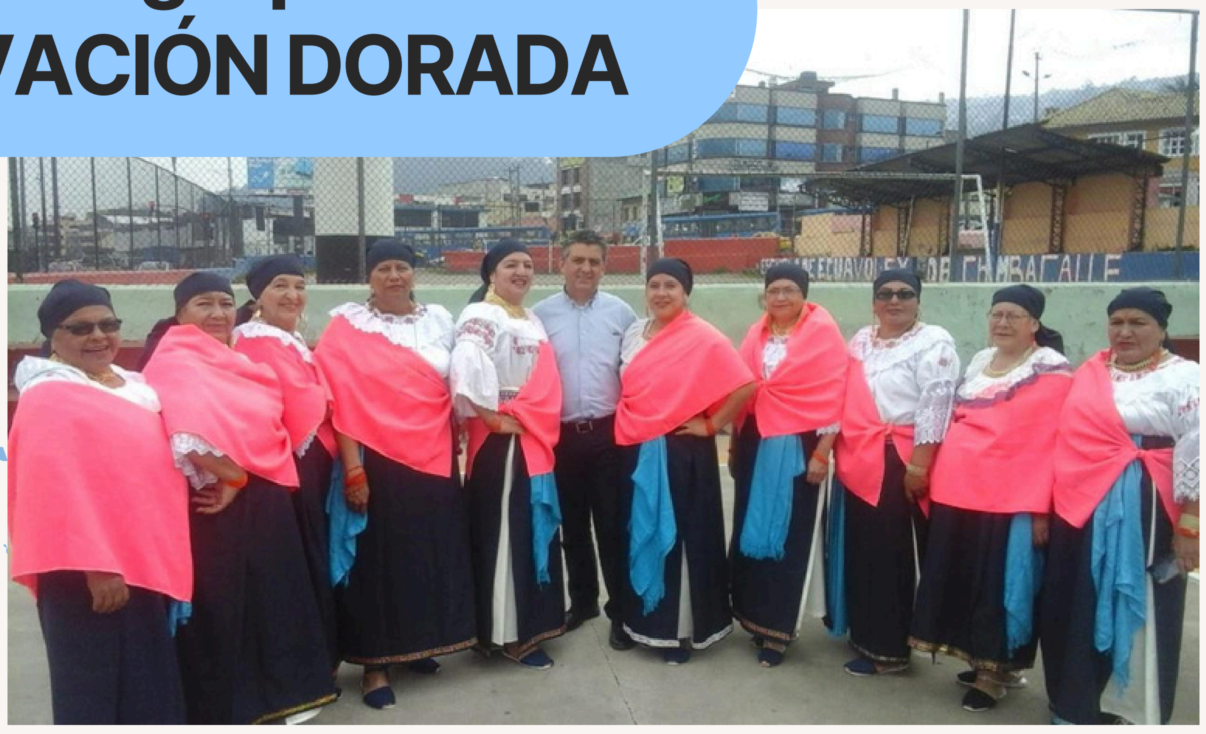

Organizado por:

PUCE | ISP

UNIVERSITAS Miguel Hernández

erc European Research Council Established by the European Commission
