# Supplementary material for: Co-creating community support strategies for chronic disease management: insights from our experience with two community groups in southern Quito, Ecuador
Source: Health Promot Int. 2026 Aug 18;41(4):daag123. doi: 10.1093/heapro/daag123 (PMC13482984; doi:10.1093/heapro/daag123)
Supplement: Supplementary file 1 [file daag123_supplementary_data.zip › SF_Tables and figures_Figure B.pdf]

Soluciones priorizadas – TOP 10

1. Creación de una **comisión comunitaria de la salud:** identificar personas con escaso soporte familiar, concienciar a la familia sobre las necesidades de las personas con diabetes, proporcionar acompañamiento a las citas médicas, evaluar necesidades en el hogar y organizar actividades de recaudación de fondos.
2. Organización de **talleres de cocina nutricional** que incluyen recetas económicas y elaboración de platos tradicionales y saludables
3. Creación de un **recetario con platos tradicionales y saludables** para compartir recetas nutritivas, incluyendo recetas adecuadas para enfermedades como la diabetes y la hipertensión
4. Creación de un **banco de medicamentos** sin ánimo de lucro para ceder medicinas a las personas del grupo que lo necesiten
5. Organización de **actividades de colaboración intergeneracional**, a los jóvenes se les enseña compostaje, siembra, recolección y recetas saludables, a cambio de ayudar a las personas adultas con aprendizaje de uso de redes sociales.
6. **Concurso de cocina saludable** con recetas tradicionales para recuperar comidas como el chapo, el morocho, la chuchuca, etc. y así fomentar de nuevo su preparación
7. **Solicitud de profesionales de atención psicológica** al centro de salud para que se organicen conversatorios de forma regular (cada dos semanas) con los clubs de pacientes de enfermedades crónicas
8. Recolección de firmas en la comunidad para **solicitar ferias de salud y brigadas médicas** para atender e informar sobre prevención y manejo de enfermedades crónicas
9. **Colaboración entre organizaciones barriales** con intercambios programados de actividades conjuntas de forma regular, incrementando la capacitación de los líderes y lideresas de la comunidad.
10. Petición al centro de salud de recibir **charlas y talleres sobre la diabetes** en la comunidad para mejorar la comprensión de las enfermedades crónicas.

erc
European Research Council
Established by the European Commission

TERECO
Creación

Tejiendo Redes Comunitarias para la Diabetes

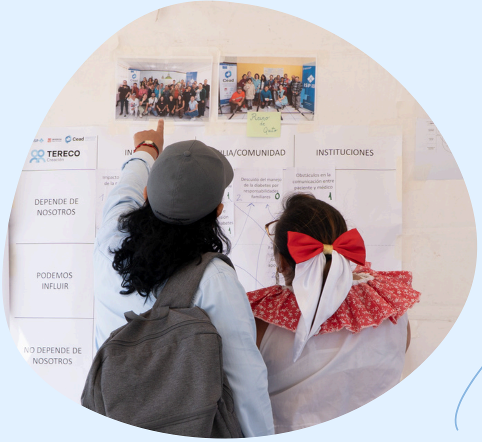

Investigación Acción-Participativa en el sur de Quito

Julio 2024

Cead
PUCE | ISP

@ceadproject
www.ceadproject.eu

¿Qué sabemos?

Vivir con **enfermedades crónicas** como la diabetes tipo 2 no es fácil. Requiere cambios en la alimentación, la actividad física, la medicación y el automanejo.
El **apoyo de la familia y el entorno** es fundamental, y la comunidad es un **activo en salud** muy importante.

¿Cuál fue el objetivo?

Diseñar **estrategias** con la comunidad para mejorar el manejo de la diabetes tipo 2, fortaleciendo las **redes de apoyo comunitario.**

¿Cómo lo hicimos?

La **comunidad** desempeñó un **papel clave**, convirtiéndose en el **equipo investigador** como expertos/as de su propio entorno, conociendo mejor que nadie los recursos disponibles y las necesidades más urgentes.

1 NOMBRAR LOS PROBLEMAS

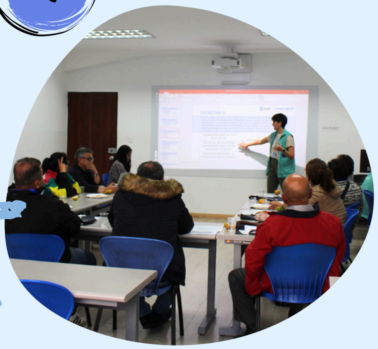

El grupo motor, compuesto por líderes/as de la comunidad, nombró las diferentes problemáticas relacionadas con el manejo de la diabetes que habían surgido a lo largo de la investigación CEAD.

2 FLUJOGRAMA

Se colocaron los problemas en una matriz según el nivel de control y el ámbito (individuo, familia/comunidad o institución).

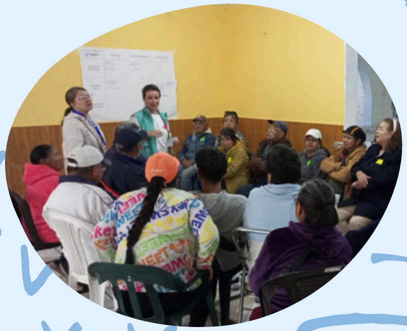

3 ÁRBOL DE PROBLEMAS

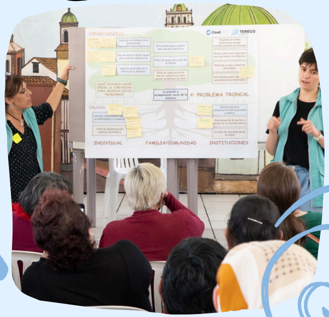

Se propusieron posibles soluciones o estrategias para abordar las problemáticas identificadas.

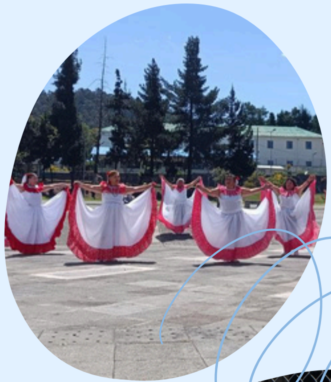

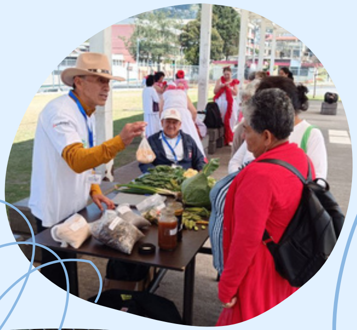

4 PRIORIZACIÓN SOLUCIONES

Se votaron las mejores propuestas, obteniendo un listado de estrategias priorizadas.

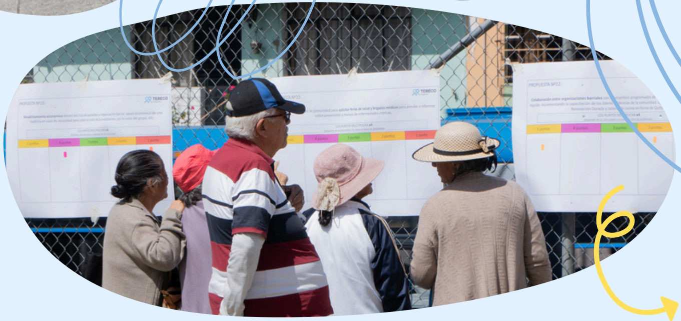

Juntos y juntas, co-creamos soluciones para fortalecer a toda la comunidad.
