# Supplementary material for: Co-creating community support strategies for chronic disease management: insights from our experience with two community groups in southern Quito, Ecuador
Source: Health Promot Int. 2026 Aug 18;41(4):daag123. doi: 10.1093/heapro/daag123 (PMC13482984; doi:10.1093/heapro/daag123)
Supplement: Supplementary file 1 [file daag123_supplementary_data.zip › SF_Tables and figures_Figure C.pdf]

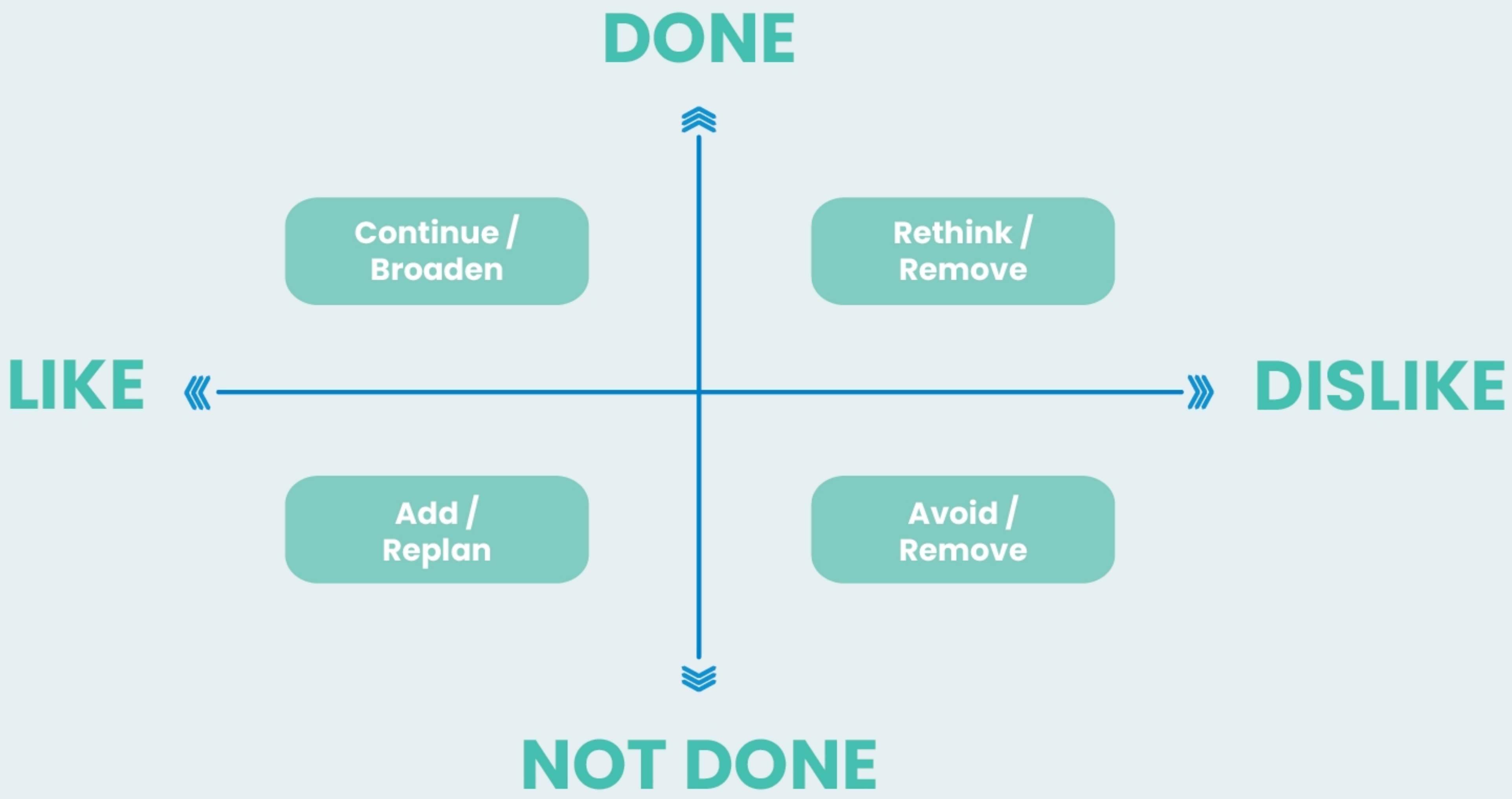
DONE
Continue / Broaden
Rethink / Remove
LIKE
DISLIKE
Add / Replan
Avoid / Remove
NOT DONE
