# Supplementary material for: Co-creating community support strategies for chronic disease management: insights from our experience with two community groups in southern Quito, Ecuador
Source: Health Promot Int. 2026 Aug 18;41(4):daag123. doi: 10.1093/heapro/daag123 (PMC13482984; doi:10.1093/heapro/daag123)
Supplement: Supplementary file 1 [file daag123_supplementary_data.zip › SF_Tables and figures_Figure D.pdf]

PROPUESTA Nº3:

TERECO Creación

Solicitud al centro de salud de profesionales de **atención psicológica** para que se organicen conversatorios de forma regular (cada dos semanas) con los clubs de pacientes de enfermedades crónicas.

LOS PUNTOS MULTIPLICAN **x2**
(depende de la comunidad)

5 puntos	4 puntos	3 puntos	2 puntos	1 punto
