# Supplementary material for: Co-creating community support strategies for chronic disease management: insights from our experience with two community groups in southern Quito, Ecuador
Source: Health Promot Int. 2026 Aug 18;41(4):daag123. doi: 10.1093/heapro/daag123 (PMC13482984; doi:10.1093/heapro/daag123)
Supplement: Supplementary file 1 [file daag123_supplementary_data.zip › SF_Tables and figures_Table A.docx]

Table A. Description of each challenge

| Scope | Challenges in T2DM | Description |
| --- | --- | --- |
| Social and Community Context | Lack of Emotional Support from Family | When family support is absent, patients often feel lonely, stressed, and even depressed. Diabetes requires changes in health behaviors (diet, exercise, medication, etc.), impacting daily life and requiring regular medical visits. The absence of family members during appointments and in daily disease management can leave patients feeling isolated and discouraged. |
|  | Social Pressure and Misunderstanding of Diabetes | Some people with diabetes feel pressured to consume unhealthy foods or drinks at social events, making it challenging to manage their disease. To avoid these situations, they may skip family or social gatherings, leading to the loss of friendships and feelings of isolation and loneliness. Many social events revolve around alcohol, which can leave abstainers feeling excluded. |
|  | Neglect of Diabetes Management Due to Family Responsibilities | Patients often neglect diabetes management due to other responsibilities. Women, who are frequently caregivers, prioritize their family’s needs, sometimes ignoring their own health. Men often neglect disease control due to economic responsibilities, work schedule conflicts, or social pressures to appear strong. |
|  | Challenges in Adapting Family Meals | Some people struggle with family members when trying to change eating habits to improve health. This may be due to customs, preferences, or a lack of awareness of the importance of a healthy diet in managing diabetes. These situations can create family tension and make it harder to adopt and maintain healthier eating habits. |
| Economic Stability | We Stop Taking Medication Because We Can’t Afford It and Feel Uncomfortable Asking for Help | Many people with diabetes need financial support from their surroundings to purchase medications. Access to diabetes medications (insulin, oral antidiabetics) depends on the healthcare system and insurance, but medication shortages in these institutions often force patients to pay out of pocket, creating inequalities in access to medications. |
|  | Poor Working Conditions for People with Diabetes | Professionals observe that work conditions are not adapted to the needs of people with diabetes or their families. Diabetic patients face difficulties balancing work with managing their condition. Economic pressure often leads to prioritizing work over health, resulting in neglect of self-care and proper disease management. This is especially relevant for men. |
| Education | Limited Knowledge About the Disease and Its Management | Limited knowledge about the disease and its management (medication, footwear, nutrition, medical check-ups, etc.) is a significant barrier for patients and their families in coping with diabetes. This issue is particularly highlighted by healthcare providers. |
|  | Lack of Digital and Technological Literacy | Many patients use the internet and social media to stay in touch with loved ones and community groups and to access information about organized activities and disease management tips. However, barriers to accessing this communication are more pronounced for older adults with diabetes who live alone, have difficulty using social networks or cannot afford internet. |
|  | Lack of Training for Community Leaders to Support Health Programs | Faith plays a significant role in diabetes care. Community leaders, especially religious ones, have great potential to spread information and promote healthy lifestyles within their groups. Involving these leaders in community health programs would help provide comprehensive support that could improve patient outcomes. |
| Healthcare System | Communication Barriers Between Patient and Doctor | Professionals note that low education levels hinder communication with patients diagnosed with type 2 diabetes, making it difficult for them to understand necessary recommendations for proper disease management, treatment adherence, and healthy habits. |
|  | Insufficient Social Investment in Healthcare | There is a perception of insufficient medical staff (with ongoing training), a lack of supplies, and necessary medications for diabetes treatment, leading to feelings of abandonment among patients and frustration among healthcare professionals. They cite limited government support for healthcare facilities as a cause. |
|  | Difficulties in Obtaining Personalized Care | There are challenges in obtaining integrated, multidisciplinary, and intersectoral care that addresses the social, economic, and healthcare needs of diabetes patients. Professionals call for individual social assessments of each patient’s situation. |
|  | Discrimination from One Sector to Another in Healthcare Access | Differences in healthcare quality between areas/centers, in terms of both staff treatment and availability of medical supplies and services, can create frustration among patients and unequal care across the population. |
