# Supplementary material for: Co-creating community support strategies for chronic disease management: insights from our experience with two community groups in southern Quito, Ecuador
Source: Health Promot Int. 2026 Aug 18;41(4):daag123. doi: 10.1093/heapro/daag123 (PMC13482984; doi:10.1093/heapro/daag123)
Supplement: Supplementary file 1 [file daag123_supplementary_data.zip › SF_Tables and figures_Table B.docx]

Table B. Voting systematization

| **Display order** | **Proposal** | **5 p.** | **4 p.** | **3 p.** | **2 p.** | **1 p.** | **CF*** | **Total points** |
| --- | --- | --- | --- | --- | --- | --- | --- | --- |
| 1 | Creation of a community health commission with activities such as identifying people with limited family support, raising family awareness of the needs of people with diabetes, providing accompaniment to medical appointments, assessing home needs, and organizing fundraising activities. | 28 | 3 | 6 | 6 | 5 | x6 | 1122 |
| 2 | Organization of nutritional cooking workshops that include affordable recipes and preparation of traditional, healthy dishes. | 7 | 27 | 1 | 1 | 8 | x6 | 936 |
| 3 | Request for psychological care professionals at the health center to hold regular (bi-weekly) conversations with chronic disease patient clubs. | 3 | 11 | 9 | 7 | 0 | x2 | 200 |
| 4 | Request to the health center for talks and workshops on diabetes within the community to improve understanding of chronic diseases. | 0 | 3 | 14 | 7 | 5.5 | x2 | 147 |
| 5 | Organization of intergenerational collaboration activities, where youth learn composting, planting, harvesting, and healthy recipes in exchange for helping older adults with social media skills. | 3 | 1 | 8 | 5 | 8 | x6 | 366 |
| 6 | Promotion of urban gardening among youth with school visits and the reintroduction of natural sciences in schools to increase youth knowledge on ecological and healthy eating. | 3 | 1 | 5 | 5 | 4 | x2 | 96 |
| 7 | Training community health agents to spread information on diabetes and other preventable diseases, including outreach to families of people with these chronic illnesses. | 0 | 0 | 2 | 6 | 2 | x2 | 40 |
| 8 | Creation of a non-profit medication bank to provide medicine to group members in need. | 12 | 2 | 4 | 5 | 9.5 | x4 | 398 |
| 9 | Healthy cooking contest with traditional recipes to revive dishes like “chapo”, “morocho”, and “chuchuca”, promoting their preparation. | 0 | 5 | 5 | 10 | 6 | x6 | 366 |
| 10 | Economic sponsorship within the patient club or neighborhood organization: financial support from a "godparent" for those in need, covering medication costs, group fees, etc. | 1 | 2 | 4 | 1 | 2 | x4 | 116 |
| 11 | Collection of community signatures to request health fairs and medical brigades to provide care and information on chronic disease prevention and management. | 2 | 3 | 6 | 3.5 | 1 | x4 | 192 |
| 12 | Collaboration between neighborhood organizations with regularly scheduled joint activities, enhancing the training of community leaders. Example: Handicraft workshops in Renovación Dorada and cooking workshops in Reino de Quito. | 2 | 4.5 | 0 | 6 | 2 | x4 | 168 |
| 13 | Dissemination of information on diabetes prevention and care through neighborhood WhatsApp groups and informative posters in community centers and common areas to reach people without social media. | 1 | 4.5 | 0 | 3 | 3 | x4 | 128 |
| 14 | Creation of a recipe book with traditional and healthy dishes to share nutritious recipes, including those suitable for conditions like diabetes and hypertension. | 8 | 5 | 2 | 1 | 3 | x6 | 426 |
| 15 | Organization of social theater workshops to strengthen community bonds and perform skits depicting the daily lives of people with diabetes, raising awareness of their needs. | 0 | 0 | 1 | 2 | 4 | x6 | 66 |
| 16 | Organization of conversations to share difficulties in managing diabetes, creating a safe space for advice and emotional support within the group. | 1 | 0 | 4 | 1 | 4 | x4 | 92 |

*Correction factor
