# Supplementary material for: Co-creating community support strategies for chronic disease management: insights from our experience with two community groups in southern Quito, Ecuador
Source: Health Promot Int. 2026 Aug 18;41(4):daag123. doi: 10.1093/heapro/daag123 (PMC13482984; doi:10.1093/heapro/daag123)
Supplement: Supplementary file 1 [file daag123_supplementary_data.zip › Supplementary caption.docx]

**Supplementary files;**

SF_Flowchart systematization

Figure A. Invitation flyer to promote the prioritization event (illustrative)

Figure B. Brochure was developed, summarizing prioritized proposals and co-creation process (illustrative)

Figure C. Tetralemma matrix used for guiding the evaluation

Figure D. Example of proposal display with votes (illustrative)

Table A. Description of each challenge

Table B. Voting systematization
